# A Promising DNA Methylation Signature for the Triage of High-Risk Human Papillomavirus DNA-Positive Women

**DOI:** 10.1371/journal.pone.0091905

**Published:** 2014-03-19

**Authors:** Alfred Hansel, Daniel Steinbach, Christiane Greinke, Martina Schmitz, Juliane Eiselt, Cornelia Scheungraber, Mieczyslaw Gajda, Heike Hoyer, Ingo B. Runnebaum, Matthias Dürst

**Affiliations:** 1 Department of Gynaecology, Jena University Hospital, Jena, Germany; 2 IMSID, Jena University Hospital, Jena, Germany; 3 Institute of Pathology, Jena University Hospital, Jena, Germany; Albert Einstein College of Medicine, United States of America

## Abstract

High-risk human papillomavirus (hrHPV)-DNA testing is frequently performed parallel to cytology for the detection of high-grade dysplasia and cervical cancer particularly in women above 30 years of age. Although highly sensitive, hrHPV testing cannot distinguish between HPV-positive women with or without clinically relevant lesions. However, in principle discrimination is possible on the basis of DNA methylation markers.

In order to identify novel DNA regions which allow an effective triage of hrHPV-positive cases, hypermethylated DNA enriched from cervical cancers was compared with that from cervical scrapes of HPV16-positive cases with no evidence for disease by CpG island microarray hybridization. The most promising marker regions were validated by quantitative methylation-specific PCR (qMSP) using DNA from archived cervical tissues and cervical scrapes. The performance of these markers was then determined in an independent set of 217 hrHPV-positive cervical scrapes from outpatients with histopathological verification.

A methylation signature comprising the 5′ regions of the genes *DLX1*, *ITGA4*, *RXFP3*, *SOX17* and *ZNF671* specific for CIN3 and cervical cancer (termed CIN3+) was identified and validated. A high detection rate of CIN3+ was obtained if at least 2 of the 5 markers were methylated. In the subsequent cross-sectional study all cervical carcinomas (n = 19) and 56% (13/23) of CIN3 were identified by this algorithm. Only 10% (11/105) of hrHPV-positive women without histological evidence of cervical disease were scored positive by the methylation assay. Of note is that the detection rate of CIN3 differed between age groups. Eight of nine CIN3 were detected among women ≥30 years of age but only five of fourteen among <30 year old group (p = 0.03). The specificity for CIN3+ in the older age group was 76.6% (95% CI 65.6–85.5%). Clinical validation studies are required to determine the usefulness of these novel markers for triage after primary hrHPV testing in a cervical cancer screening setting.

## Introduction

Cervical cancer is one of the most frequent cancers among women worldwide with 528.000 newly diagnosed cases and 266.000 deaths in the year 2012 [1]. Primary prevention through vaccination or secondary prevention by eradication of precursor lesions are the most effective strategies to reduce the cancer burden.

The cytology-based Pap test is the most widely used cervical cancer screening method [2]. However, in a study including more than 60.000 women Pap smear screening failed to detect about half of the CIN2, CIN3 and cancer cases (referred to as CIN2+ cases). Moreover, only 20% of the women diagnosed with an abnormal Pap smear had CIN2+ [3]. Screening for high-risk human papillomavirus (hrHPV) is thus discussed as an attractive alternative to the subjective Pap smear cytology [4]; [5]. In the USA, both HPV testing and Pap smear are recommended for screening women aged >30 years [6]. In fact, since infection with hrHPV is a prerequisite for the development of cervical cancer, a negative HPV result excludes CIN2+. A positive HPV result, however, has insufficient specificity since it does not discriminate between cancer-relevant lesions (CIN2+) and transient, clinically irrelevant hrHPV infections (≤CIN1) [3]; [7]. Overall referral rates to colposcopy are high in a screening setting. Therefore diagnostic tests which can distinguish between women who are only transiently infected with hrHPV and those with cervical disease are required. Cytology applied as a reflex test to hrHPV-positive women is considered an appropriate tool [8], but in the light of a positive hrHPV result minor cellular abnormalities might be over-interpreted, thereby lowering specificity [7]. To overcome these shortcomings, double-immunostaining for HPV-transformed cells which characteristically express both p16 and the proliferation marker Ki67, was proposed. In a recent study double-staining for p16/Ki67 (CINtec plus) showed a sensitivity for CIN3+ of 93%, but a specificity of only 46% due to a high detection rate of subjects without histology-confirmed disease [9]. Further, molecular biomarker analyses yielding more objective results than cytology are currently being evaluated. Several studies have shown that abnormalities of the genome and the epigenome underlie cancer [10]. Indeed, DNA hypermethylation of the promoter and 5′ regions of tumour suppressor genes was shown to be an early event in carcinogenesis [11]. In this regard, DNA hypermethylation is very attractive for diagnostics, since it can easily be assessed by molecular methods [12]. Subsequently, numerous DNA regions hypermethylated in cancer tissue have been proposed as markers for cervical cancer diagnostics (for an overview see [13]). Several research groups have already provided proof of principle for applying methylation-specific PCR using DNA from cervical scrapes [14], [15] or cervical lavages [16]. Nevertheless the search for optimal methylation markers for reflex-testing hrHPV-positive women is still ongoing.

In the present study we have used a novel approach to identify highly discriminating DNA methylation markers. The methylation profile of epithelial cells derived from cervical scrapes of HPV16-positive cases with no evidence for disease was compared to that of cervical carcinoma biopsies. For this purpose methylated DNA was enriched by a methylated-CpG island recovery assay (MIRA) and subsequently used in hybridization experiments using genome-wide CpG island microarrays. The performance of the identified and validated markers was then evaluated in an independent set of cervical scrapes from women attending our outpatient colposcopy clinic.

## Results

### Genome-wide DNA methylation analysis reveals differentially methylated CpG islands

**Figure 1:**
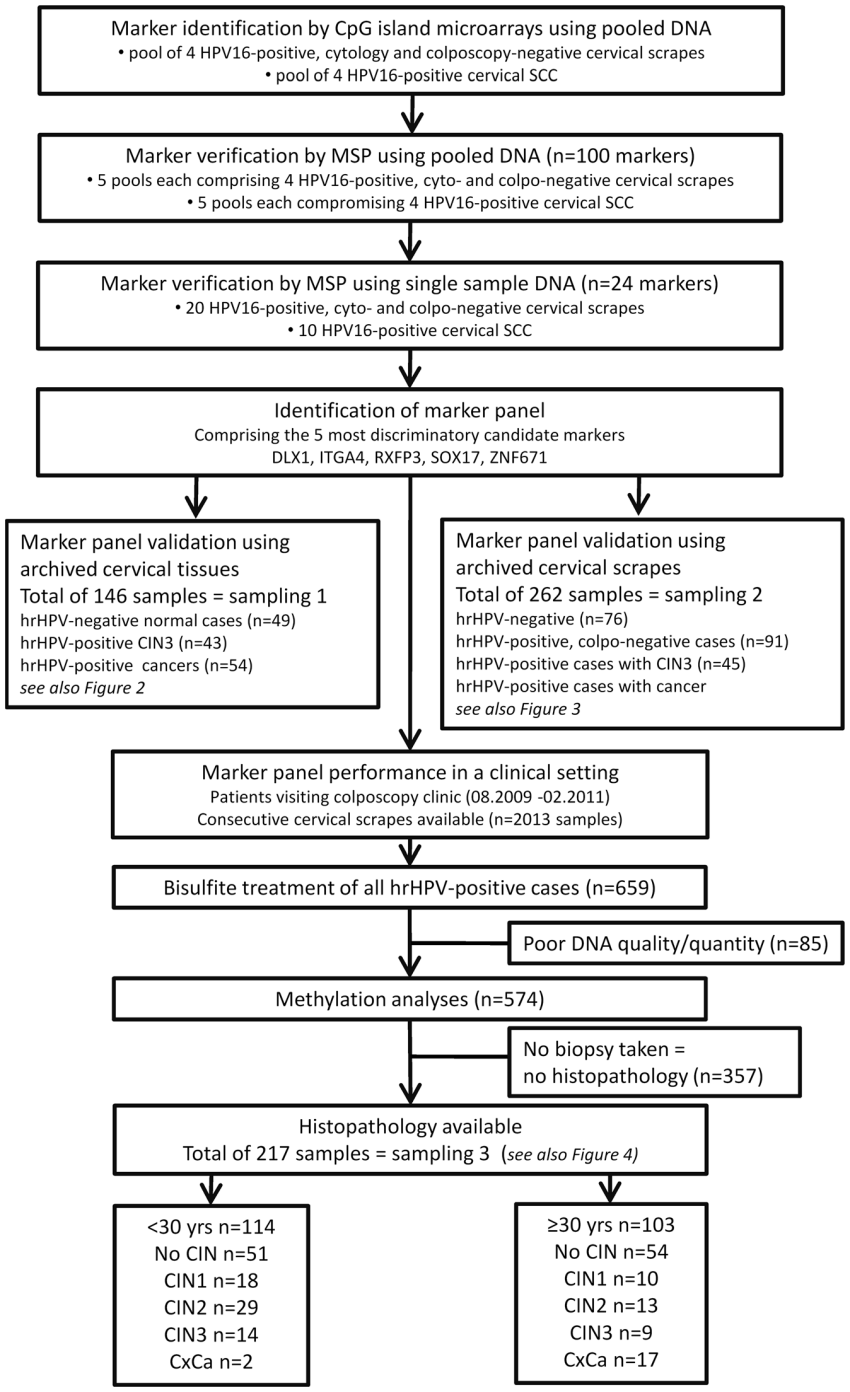
Flowchart showing all steps performed for marker identification, verification and validation. SCC: squamous cell carcinoma; CIN: cervical intraepithelial neoplasia; CxCa: cervical carcinoma; MSP: methylation specific PCR; hrHPV: high-risk HPV.

CpG island microarray experiments were performed in order to detect DNA regions exclusively methylated in cancer tissue. These tiling arrays comprise ca 237.000 probes covering most CpG islands present in the human genome. The analysis revealed several DNA regions with distinct differences in the methylation level between DNA enriched from cervical scrapes from HPV16-positive women without disease and DNA enriched from cancer tissue. Of the CpG islands with at least eight times higher fluorescence level for cancer DNA, 100 marker regions gave very weak hybridization signals with DNA from scrapes, but high signals with tumour DNA for 3 consecutive probe spots (Figure 1). For these regions, methylation-specific PCRs (qMSP) were established, and tested on DNA pools each containing equal amounts of four different sample DNAs. Overall, five pools comprising tumour DNA and five pools comprising DNA from cervical scrapes of HPV-positive women having no cytologic and colposcopic evidence for cervical disease ( = controls) were used. Of the 100 CpG regions tested 24 showed no methylation-specific amplification for any control pools but positive results for at least three tumour pools (highlighted in colour in Table S1). These 24 CpG regions were then further validated using DNA from single samples. Twenty cervical scrapes from HPV-positive women without cervical disease as well as 10 cancer tissue samples were used for this purpose. Five marker regions could discriminate best between normal and tumour samples. They were positive for at least five cancer tissue samples and positive for no more than two of the 20 control samples (Table 1 and Table S1). These five marker regions comprising DLX1, ITGA4, RXFP3, SOX17, and ZNF671 were subsequently tested in further qMSP experiments.

**Table 1:** DNA marker regions identified by genome-wide methylation array analysis.

Gene region	Chromosome	Base position
DLX1	Chr 2	172,945,912–172,946,212
ITGA4	Chr 2	182,321,762–182,323,029
RXFP3	Chr 5	33,936,169–33,938,309
SOX17	Chr 8	55,370,171–55,372,525
ZNF671	Chr 19	58,238,586–58,239,028

Nucleotide positions are given according to the hg19 genome annotation.

### DNA methylation status of the marker regions DLX1, ITGA4, RXFP3, SOX17, and ZNF671 in cervical tissue specimens

**Figure 2:**
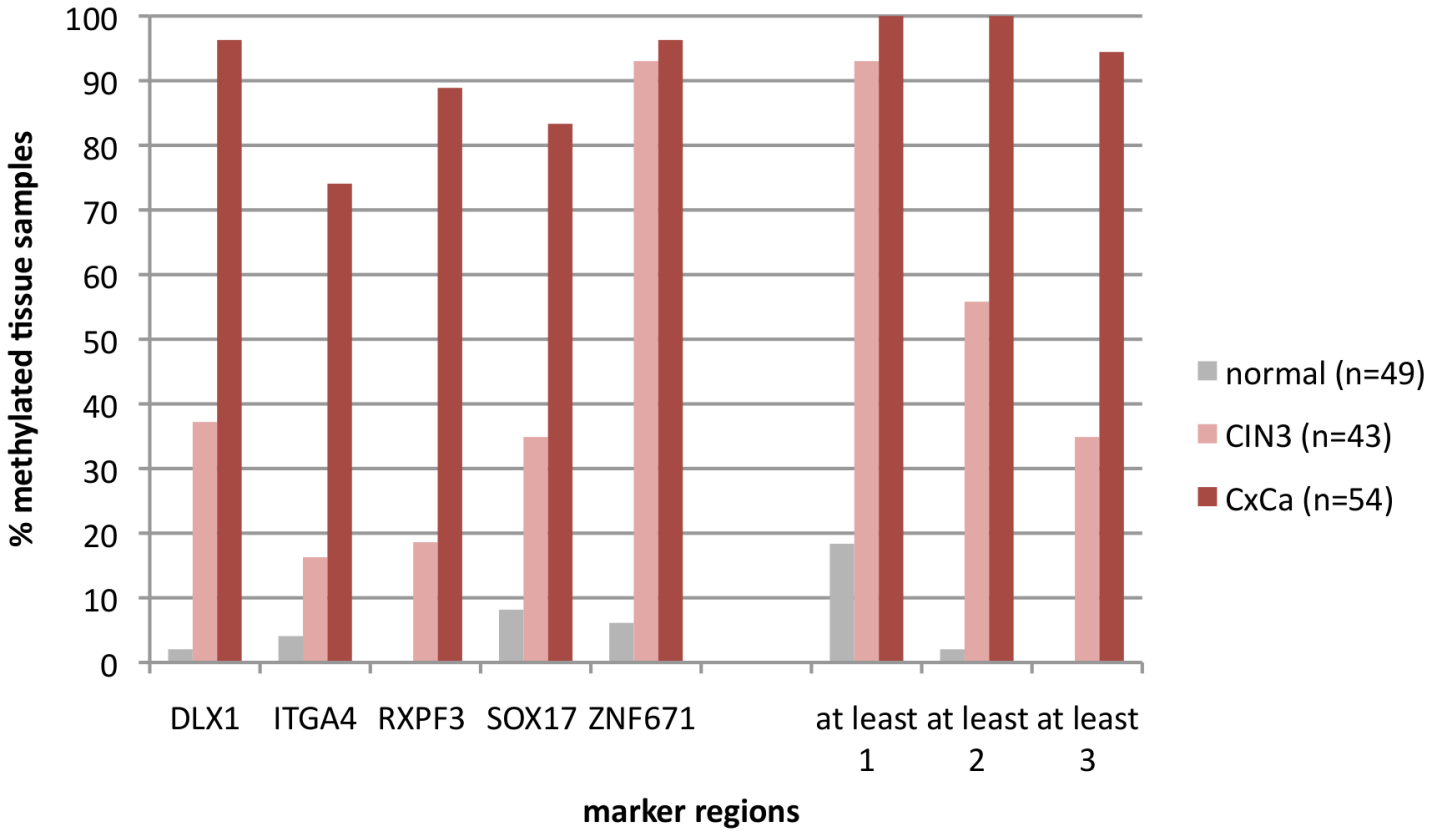
Single-marker qMSP experiments with bisulfite-treated DNA isolated from tissue sections (sampling 1) of histologically confirmed normal cervical epithelium (n = 49 cases), CIN3 (n = 43) and cervical cancer (CxCa; n = 54). All cancers were squamous cell cancers. For HPV-genotyping see Table S2.

Tissue specimens were available from 146 women (Figure 1, sampling 1). DNA isolated from 49 tissue samples with no histological evidence for CIN (normal), from 43 samples diagnosed as CIN3 and from 54 cervical cancer tissues was bisulfite-treated and used in qMSP experiments for the five marker regions DLX1, ITGA4, RXFP3, SOX17, and ZNF671. In these qMSPs the individual marker regions showed DNA methylation rates for CIN3 between 14 and 93% and for carcinoma between 74 and 96% (Figure 2). The ZNF671 marker region showed the highest detection rates for both, CIN3 (40 of 43 samples; 93%) and carcinoma samples (52 of 54 samples; 96%). Moreover, this marker region was only rarely methylated in normal tissue (3 of 49 samples; 6%). Considering an algorithm in which at least two of the five marker regions need to be methylated in order to be scored positive, 24 of 43 CIN3 (55%), all 54 carcinoma samples (100%) but only one of 49 normal hrHPV-negative tissues (2%) would be detected (Figure 2).

### DNA methylation of the five marker regions in cervical scrapes correlates with underlying histopathologically confirmed disease

**Figure 3:**
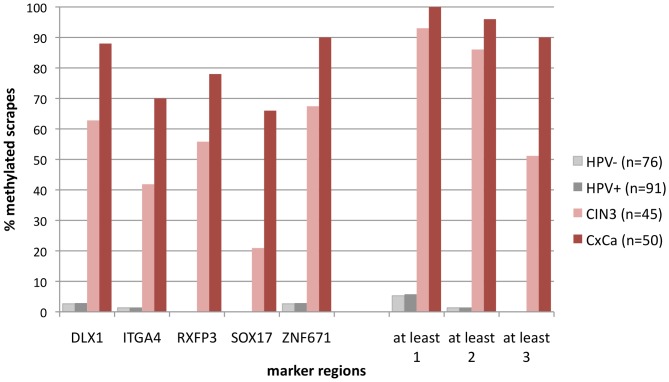
Single-marker qMSP experiments with bisulfite-treated DNA isolated from cervical scrapes (sampling 2) of hrHPV-negative women (n = 76), hrHPV-positive women with normal colposcopy (n = 91), and hrHPV-positive women with histologically confirmed CIN3 (n = 45) and cervical cancer (CxCa; n = 50). By using the algorithm “at least 2 of 5 markers” need to be methylated in order to score the sample methylation positive one of 8 adenocarcinoma and one of 42 SCC were false negative.

The promising results obtained from tissue biopsies were validated using DNA from a selected subset of 262 archived cervical scrapes (Figure 1, sampling 2). As was the case for the tissues, qMSP analyses for the ZNF671 marker region showed the highest detection rates for cervical scrapes with underlying CIN3 (67%) and carcinoma (90%) (Figure 3). Overall the methylation rates for the single marker regions ranged from 21 to 67% for CIN3, and from 66 to 90% for carcinoma. At least one of five markers was methylated in 93% of CIN3 and all CxCa samples, but in less than 6% of the control samples from disease-free HPV-negative and HPV-positive women. At least two of five markers were methylated in 86% and 96% of CIN3 and CxCa samples, and in less than 2% of the control samples from disease-free HPV-negative and HPV-positive women, respectively.

### Diagnostic performance of the marker panel in a cross-sectional study

In order to demonstrate the diagnostic potential of the markers for the detection of CIN3+ among women who tested hrHPV-positive, a cross-sectional study was performed for patients referred to our outpatient colposcopy clinic (Figure 1).

**Figure 4:**
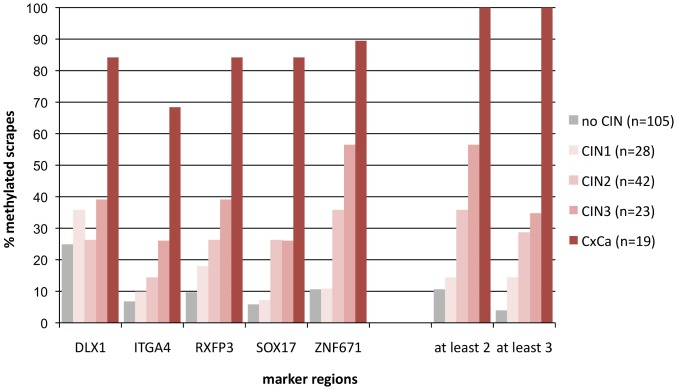
Diagnostic performance of the DNA methylation marker panel for hrHPV-positive cervical scrapes in relation to histopathology (sampling 3). For HPV-genotyping see Table S4.

Overall 2013 patients were examined by colposcopy. Biopsies were taken if indicated. Of all patients cervical scrapes were taken for cytology and hrHPV testing by the GP5+/6+ PCR-EIA assay [17]. 659 (32.7%) patients were hrHPV-positive. DNA isolated from all hrHPV-positive cases was bisulfite treated and analysed by qMSP for the marker regions DLX1, ITGA4, RXFP3, SOX17, and ZNF671. Eighty-five cases could not be evaluated because of insufficient amounts of DNA or poor quality (ACTB Ct values >34). For 217 patients histopathological data were available. This group is referred to in Figure 1 as sampling 3. The age ranged from 18 to 81 (mean 32.8) years, 48% of them were at least 30 years old. 105 of these women had no histological evidence for CIN, 28 were diagnosed with CIN1, 42 with CIN2, 23 with CIN3 and 19 with cervical cancer (18 SCC and 1 adenocarcinoma). The DNA methylation rates obtained for the single marker regions ranged from 26% to 56% for women with CIN3 and from 68% to 89% for cancer patients (Figure 4). Highest DNA methylation rates were again observed for the ZNF671 marker region. At least 2 of 5 markers were methylated in all 19 cancer cases and in 13 of 23 (56%) CIN3 cases. Only 11 of the 105 (10%) patients diagnosed as having no CIN were scored positive under these conditions. A bias resulting from varying DNA quality in the different histological groups is unlikely, since the mean Ct values for ACTB differed by a maximum of 0.73 between the groups no CIN, CIN1, CIN2, CIN3 and CxCa.

Stratifying the data by age revealed interesting differences in the detection rate of CIN. Among women histopathologically diagnosed with CIN3, 36% (5/14) were detected by at least 2 markers among women <30 years of age, but 89% (8/9) among ≥30 year old group (Table 2). The two proportions differ significantly (p = 0.03). These differences are still observed even when altering the algorithm to at least 3 of 5 markers to be methylated in order to be scored positive (Table 2, Table S3). However, under such stringent conditions the overall detection rate of CIN3 would only be 35% which is clearly too low in a triage setting. Accordingly, the diagnostic performance of the methylation marker panel is best for the algorithm “at least 2 of 5”. Under these conditions the assay has a statistically significant higher sensitivity for CIN3+ of 96.2% (95% CI 80.4–100%) in women ≥30 years of age as compared to the younger women (p<0.01) (Table 3). Specificity was 76.6% (95% CI 65.6–85.5%) in women ≥30 years which is about 10% lower than in younger ages (p = 0.07) (Table 3, row a). Moreover, the average Ct values for all positive cases according to their histological classification did not differ among the age groups (Figure S1). However, sampling 3 is a rather untypical cohort since it comprises 19 cancers and 23 CIN3. We have therefore also calculated the sensitivity and specificity for CIN2/3 and CIN3 without the cancer cases. Under these conditions the sensitivity for CIN2/3 and CIN3 for women ≥30 years of age was 77.3% (95% CI 54.6–92.2%) and 88.9% (95% CI 51.8–99.7%), respectively. Specificity remained unaltered and was 85.9% (95% CI 75.0–93.4%) and 76.6% (95% CI 65.6–85.5%), respectively (Table 3, row b).

**Table 2:** DNA methylation analysis using bisulfite-treated DNA from cervical scrapes of 217 women with histologically confirmed cervical disease status (sampling 3).

	Women <30 (n = 114)	Women ≥30 (n = 103)	Total (n = 217)
**No of markers: ≥2**	Methylation-positive/total number n (% methylated; 95% CI)		
no CIN	5/51 (9.8%; 3.3–21.4%)	6/54 (11.1%; 4.2–22.6%)	11/105 (10.5%; 5.4–18.0%)
CIN1	1/18 (5.6%; 0.1–27.3%)	3/10 (30.0%; 6.7–65.3%)	4/28 (14.3%; 4.0–32.7%)
CIN2	6/29 (20.7%; 8.0–39.7%)	9/13 (69.2%; 38.6–90.9%)	15/42 (35.7%; 21.6%–52.0%)
CIN3	5/14 (35.7%; 12.8–64.9%)	8/9 (88.9%; 51.8–99.7%)	13/23 (56.5%; 34.5–76.8%)
CxCa	2/2 (100%; 22.4–100%)	17/17 (100%; 83.8–100%)	19/19 (100%; 85.4–100%)
**No of markers: ≥3**	Methylation-positive/total number n (% methylated; 95% CI)		
no CIN	1/51 (2.0%; 0.0–10.5%)	3/54 (5.6%; 1.2–15.4%)	4/105 (3.8%; 1.1–9.5%)
CIN1	1/18 (5.6%; 0.1–27.3%)	3/10 (30.0%; 6.7–65.3%)	4/28 (14.3%; 4.0–32.7%)
CIN2	5/29 (17.2%; 5.9–35.8%)	7/13 (53.9%; 25.1–80.1%)	12/42 (28.6%; 15.7–44.6%)
CIN3	2/14 (14.3%; 1.8–42.8%)	6/9 (66.7%; 29.9–92.5%)	8/23 (34.8%; 16.4–57.3%)
CxCa	2/2 (100%; 22.4–100%)	17/17 (100%; 83.8–100%)	19/19 (100%; 85.4–100%)

Total number and percentage, with 95% confidence intervals, of samples which were methylation-positive for at least two (upper part of table) or three (lower part of table) of five DNA marker regions.

**Table 3:** Diagnostic performance of the methylation marker panel (sampling 3) with (a) and without (b) cancer cases.

	Sensitivity (%)	Specificity (%)	Sensitivity (%)	Specificity (%)
	a) CIN 2+	CIN 2+	a) CIN 3+	CIN 3+
	b) CIN2/3		b) CIN 3	
both age groups	a) 56.0 (44.7–66.8)	88.7 (82.1–93.5)	a) 76.2 (60.5–87.9)	82.9 (76.4–88.1)
	b) 43.1 (30.8–56.0)		b) 56.5 (34.5–76.8)	
women <30 years	a) 28.9 (16.4–44.3)	91.3 (82.0–96.7)	a) 43.8 (19.8–70,1)	87.8 (79.6–93.5)
	b) 25.6 (13.5–41.2)		b) 35.7 (12.8–64.9)	
women ≥30 years	a) 87.2 (72.6–95.7)	85.9 (75.0–93.4)	a) 96.2 (80.4–100)	76.6 (65.6–85.5)
	b) 77.3 (54.6–92.2)		b) 88.9 (51.8–99.7)	
p-value	a) <0.01	0.41	a) <0.01	0.07
	b) <0.01		b) 0.03	

To be scored methylation positive if at least 2 of 5 markers were methylated. P-values refer to Fisher exact test, comparing test performance by age-group.

## Discussion

There is increasing evidence that methylated DNA marker regions are an appropriate triage tool for hrHPV-positive women. These marker regions, often referred to as methylation marker panels, are discriminatory for women with cervical disease [7], [16], [18]. However, none of the markers are absolutely specific for high grade disease. Because of this shortcoming, receiver operating curve (ROC) analyses were performed in most studies in order to define threshold values for individual markers or marker panels which allowed the best trade-off between sensitivity and specificity. To overcome this dilemma our strategy for the identification of novel marker regions was based on the comparison of HPV16-positive cervical scrapes from women with no evidence for disease and biopsy tissue of women with cervical carcinoma. By this approach we hypothesized that the number of false-positive cases in a triage setting could be reduced. Indeed, none of the five marker regions identified in our study were methylated in more than 5% of hrHPV-positive cervical scrapes from disease-free patients used for analytical validation (Figure 3). Accordingly, the percentage was even lower (<2%) when any two of the five markers were required to be methylated for the sample to be scored positive. Despite this low rate of false positives, the detection rate of CIN3 and cancer was high, reaching 86% and 96% respectively (Figure 3). Interestingly, when analysing the diagnostic performance of our marker panel in a subsequent independent cross-sectional study, the rate of false positives among patients without histopathological evidence for CIN based on the algorithm “two of five markers” was 10% (Figure 4). Unexpectedly, a methylation rate of 10% was also observed for the 357 cases who had no biopsy taken (Figure 1 and data not shown). One explanation for this high positive rate among the latter group may be that despite colposcopic abnormalities biopsy may not always have been taken e.g. in case of late term pregnancy. Moreover, among the 217 cases of whom histopathology was available, biopsy may not always have been taken at the *punctum maximum*. Thus, prevalent lesions could have been missed and the methylation assay, which analyses cells scraped from the entire cervix, would be falsely interpreted as false-positive. In contrast, for marker validation (sampling 2, Figure 3) all hrHPV-positive cases without evidence for disease were based on normal colposcopy without biopsy. Of interest is also the observation that our methylation panel detected high grade disease at different rates dependent on age. The detection rates in women <30 and ≥30 years of age for CIN2 was 21% and 69% and that for CIN3 was 36% and 89%, respectively (Table 2). Possibly this data reflects the natural history of CIN which may differ considerably among both age groups. Clearly, not all CIN3 inevitably progress to invasive cancer [19]. Moreover, spontaneous regression of CIN, even high grade CIN, may be higher in young women. This assumption would be in line with the observed high prevalence rate of high grade CIN in young women [20]. It is tempting to speculate that our marker panel may also be of prognostic relevance. This hypothesis will have to be investigated in longitudinal studies. In analogy to sampling 1, we have also HPV-genotyped the scrapes of the cross-sectional study (Table S4). A correlation between HPV-type and age groups is not evident. However, there is a trend that independent of age, HPV16 is more prevalent in methylation-positive cases than methylation-negative cases. This applies to all disease groups, except CIN2 ≥30 years.

Our marker panel achieved a remarkably high sensitivity and specificity for CIN3+ in the cross-sectional study. For women ≥30 years of age, sensitivity and specificity for CIN3+ was 96.2% (95% CI 80.4–100%) and 76.6% (95% CI 65.6–85.5%), respectively (Table 3, row a). However, our study has several limitations. It is primarily explorative in design and does not fulfil the criteria of a clinical validation study. Nevertheless, it does provide a first impression of the diagnostic potential of the marker panel. The potential diagnostic performance was explored using a consecutive series of cervical scrapes from women visiting our outpatient colposcopy clinic. For methylation analysis we included only hrHPV-positive patients who also had a biopsy taken because of colposcopic abnormalities. This cohort was therefore highly selected and was not representative of a screening population. However, the advantage of this approach was that all of the cervical scrapes evaluated underlie histopathologically diagnosed tissues which protected against verification bias. The overall sample size was chosen with respect to feasibility. We present performance indices with 95% confidence intervals to demonstrate sample size depending precision. This cohort included an untypically high number of carcinomas, but the sensitivity for CIN2/3 and CIN3 for women ≥30 years of age is still high with 77.3% (95% CI 54.6–92.2%) and 88.9% (95% CI 51.8–99.7%), respectively, even if we exclude the carcinomas (Table 3, row b). Specificity remained unaltered and was 85.9% (95% CI 75.0–93.3%) and 76.6% (95% CI 65.6–85.5%), respectively (Table 3, row b). Moreover, we have also calculated the theoretical performance of our marker panel in a screening situation. To accomplish this we projected the test performance for sampling 3 to the target population of a HPV screening study which we had conducted in Eastern Thuringia some years ago [20]. That screening population comprised 3292 women ≥30 years of age, 194 of whom were hrHPV-positive. For this scenario sensitivity, specificity, PPV and NPV were calculated to be 90.5%, 81.0%, 61.6% and 96.2%, respectively (Table 4).

**Table 4:** Projection of methylation test performance (scored as test-positive if at least 2 of 5 markers were methylated) in hrHPV positive women ≥30 years of age originated from a screening population [20].

	hrHPV positive women ≥30 years of age (target population)				
	No CIN	CIN1	CIN2	CIN3	CxCa
Distribution of disease status in target population (p)	59.4%	7.7%	7.7%	21.6%	3.6%
Proportion of methylation-positive women per group (m)	11.1%	30.0%	69.2%	88.9%	100.0%

Projected test performance for	Sensitivity	Specificity	PPV	NPV	
CIN2+ (prev = 32.9%)	85.5%	86.7%	76.0%	92.4%	
CIN3+ (prev = 25.2%)	90.5%	81.0%	61.6%	96.2%	

sensitivity = Σ (p_d_*m_d_)/Σ p_d_ specificity = Σ (p_n_*(1-m_n_))/Σ p_n_.

p - proportion of women in the target population, m - proportion of methylation-positive women per group, d - group indices diseased, n - group indices non-diseased, NPV - negative predictive value, PPV - positive predictive value, prev – prevalence.

Several other studies have identified and validated discriminatory methylated marker regions. Overmeer and colleagues used a two marker panel consisting of CADM1 (region M18) and MAL (region M1) in a cohort of 79 women visiting an outpatient colposcopy clinic and detected CIN3+ with a sensitivity of 70% and specificity of 78% [7]. In a subsequent study it was shown that the methylation levels of both markers in hrHPV-positive scrapes are related to the degree and duration of underlying cervical disease and were markedly increased in cervical cancer [21]. In another study the diagnostic performance of a panel of four markers (JAM3, EPB41L3, TERT and C13ORF18) for hrHPV-positive patients referred with abnormal Pap smear revealed a sensitivity and specificity for CIN3+ of 84% and 69%, respectively [16]. By genome-wide methylation profiling the same research group identified two further markers, COL25A1 and KATNAL2, with a similar assay performance [22].

Moreover, methylation of the HPV genome itself has also been the focus of several studies related to innovative diagnostic approaches. Early work has shown that methylation of the HPV16 genome is associated with CIN3 and cancers [23], [24]. In a most recent study increased methylation at nine CpG sites within the L1, L2 and E2/E4 region of HPV16 was associated with increased risk for prevalent and incident CIN3 compared with control specimens [25]. In line with that data, the methylation pattern of HPV31, HPV18 and HPV45 also varied significantly between women with CIN3 and women without histological and cytological evidence of high grade CIN [26]. Thus HPV DNA methylation also has the potential to serve as a specific marker for the triage of hrHPV infected women.

An association between promoter methylation and carcinogenesis has been proposed for some of the genes of the marker set described in our study. DLX1 belongs to a group of genes with homeodomains, which may function as transcription factors. CpG islands located in the DLX1 gene region were shown to be hypermethylated in astrocytomas [27] as well as in chronic lymphatic leukemia patients [28]. This gene is expressed during embyogenesis, and its product may function as a regulator of multiple signals from TGF-beta superfamily members in broad biological contexts during blood production [29]. The possible role of this gene as a tumour suppressor is not readily apparent. Likewise, SOX17 is involved in embryogenesis. Its promoter/5′ region was recently shown to be frequently methylated in oesophageal carcinomas. Experimental data support the hypothesis that loss of SOX17 removes the normal inhibition of WNT signalling and promotes oesophageal tumorigenesis [30]. Hypermethylation of the relaxin/insulin-like family peptide receptor RXFP3 promoter region was shown to be associated with microsatellite instability in endometrial carcinomas [31]. The integrin family comprises adhesion receptors that mediate both cell–extracellular matrix and cell–cell interactions. Very recently, It has been suggested that the loss of integrin alpha4 (ITGA4) expression might be associated with metastasis in several cancers [32]. Least of all is known about ZNF671 except that it belongs to a large family of zinc-finger transcription factors. Very recently it has been shown to be methylated in a specific subgroup of clear cell renal cell carcinomas, which have distinct clinicopathological phenotypes [33].

The data presented in this study demonstrate that the panel consisting of the five DNA methylation markers DLX1, ITGA4, RXFP3, SOX17, and ZNF671 may provide a useful tool for the triage of hrHPV-positive women. Currently a CE certified in vitro diagnostic test based on these marker regions is being developed. The performance of the assay will then be evaluated in a multi-centre, prospective clinical trial.

## Materials and Methods

### Cell and tissue samples

All women attending our outpatient colposcopy clinic at the Department of Gynaecology of the Jena University Hospital are routinely tested for hrHPV infection. For this purpose genomic DNA from cervical scrapes is extracted using the QIAamp DNA Mini Kit (QIAGEN, Hilden, Germany) following the instructions of the supplier. The remaining DNA is stored at −86°C and is made available for cancer research providing informed consent is given. Cervical scrapes are always taken under colposcopic guidance and if abnormalities are evident a biopsy is taken. Thus, for a subset of cervical scrapes the data for the histological correlate is also available. Moreover, whenever surgery is performed and patients' consent was provided cervical tissue biopsies are collected for bio-banking.

An overview of the samples used in this study is given in Figure 1. For genome-wide methylation profiling cervical scrapes and cervical carcinoma biopsies were used (see section MIRA and array hybridization below). Marker validation was based on two independent samplings: selected archived biopsies (sampling 1) and selected archived cervical scrapes (sampling 2). Sampling 1 comprised 49 hrHPV-negative normal cervical tissues, 43 hrHPV-positive CIN3 and 54 hrHPV-positive cervical carcinomas, all confirmed by histopathology. Sampling 2 comprised 76 hrHPV-negative scrapes, 91 hrHPV-positive scrapes of women with no colposcopic abnormalities, 45 hrHPV-positive scrapes of women with confirmed CIN3 and 50 hrHPV-positive scrapes of women with confirmed cervical carcinoma. Test performance was evaluated in a cross-sectional study using a consecutive series of cervical scrapes from women visiting our outpatient colposcopy clinic between August 2009 and February 2011 (sampling 3). Clinico-pathological data were retrieved from patient files and stored in pseudonymised form in a database to analyse test performance.

### Ethics statement

All patients provided written informed consent to use their cervical scrapes and/or biopsy material and the corresponding clinico-pathological data for molecular analyses to be conducted in our hospital. This study was approved by the ethics committee of the Friedrich Schiller University Jena (Reference numbers 2174-12/07 and 3471-06/12).

### DNA isolation, bisulfite treatment

hrHPV testing of samples was done using the GP5+/6+ PCR-EIA assay [17]. For genotyping a multiplex PCR which detects the 7 most prevalent types in cervical cancer [34] was used. Genomic DNA was isolated from cervical scrapes collected in PBS (pH 7.4) or from tissue sections (10×10 µm) using the QIAamp DNA Mini Kit (QIAGEN, Hilden, Germany) following the instructions of the supplier. DNA was bisulfite-converted using the DNA Methylation Gold kit (Zymo Research Europe, Freiburg, Germany). Concentration of the DNA was measured using a Nanodrop 1000 UV-Vis spectrophotometer (PeqLab, Erlangen, Germany). Genomic DNA from cultured CaSki cells was in vitro methylated at CpG sites using SssI CpG methyltransferase (New England Biolabs, Frankfurt, Germany) and subsequently bisulfite-treated to serve as positive control in quantitative methylation-specific PCR (QMSP).

### MIRA and Array hybridization

DNA from cervical scrapes of four HPV16-positive cases with no evidence for disease was pooled and served as control. Tumour DNA was pooled from four HPV16-positive squamous cervical carcinoma biopsies. For enrichment of methylated DNA, the genomic DNA was first sonicated to yield fragments of ca 200–500 base-pairs size. Enrichment for methylated DNA (MIRA, [35]) was performed using the MethylCollector kit according to the manufacturer's protocol (ActiveMotif, Carlsbad, USA). MIRA-enriched DNA was fluorescently labelled using the BioPrime total genomic labelling system (Invitrogen, Darmstadt, Germany). Tumour DNA was labelled with AlexaFluor 5, control DNA with AlexaFluor 3. Both DNAs were mixed and hybridized to 244 k CpG island microarrays covering ca. 27.000 CpG islands of the human genome (Agilent, Böblingen, Germany), for 40 hours following the instructions of the supplier. Arrays were scanned in a DNA microarray scanner (Model G2565B, Agilent), and data extracted using Feature Extraction Software 10.1.1.1. Results were analyzed using the Agilent Genomic Workbench software and MS EXCEL.

### Quantitative methylation-specific PCR

Quantitative methylation-specific PCR (qMSP) was performed using the FastStart Universal SYBR Master Mix (ROX) (Roche Diagnostics, Mannheim, Germany) in a 7300 system (Applied Biosystems, Darmstadt, Germany). After a 10 min period at 95°C, 40 cycles at 95°C for 15 sec, 60–64°C (depending upon primer pair used) for 20 sec and 72°C for 30 sec were run. In each PCR reaction 5 or 20 nanograms of genomic, bisulfite-treated DNA from tissue sections or cervical scrapes, respectively, were used with 2.5 picomoles of each MSP primer in a total volume of 20 µl. As quality control for bisulfite-treated DNA, a PCR using primers for the amplification of a 133 bp fragment upstream of the ACTB gene devoid of CpG dinucleotides in the primer-binding region was performed using the conditions above, with an annealing temperature of 61°C [36]. Samples were considered to be of sufficient quality if the Ct value for ACTB was ≤34 cycles. Samples were scored methylation-positive for an individual marker region, if a PCR product characterized by its typical melting curve determined directly after qMSP amplification was obtained within 40 cycles of the corresponding PCR program. The average Ct values for all positive cases (sampling 3) according to their histological classification and age are shown in Figure S1. Moreover, representative melting curves for each marker are shown in Figure S2. The sequences of all primer pairs used are provided upon request.

The sensitivity and specificity of the primers used for methylation-specific PCR detection of the five marker regions was evaluated using a serial dilution of in-vitro methylated, bisulfite-treated DNA from the cell line CaSki. The dilutions comprised 100%, 25%, 10%, 5%, 2%, 1% and 0% of methylated DNA in a background of bisulfite-treated DNA from a cervical scrape of a healthy woman. Ten ng of DNA was used for each reaction. PCRs were performed as described as above. Three independent PCR reactions were done for each primer/DNA combination. Bisulfite-specific PCR primers for ACTB were included in this experiment. The same primer pair was used for quality control of all clinical samples of the study. The results demonstrate that the primer pair for each marker region allowed the detection of 1% to 2% methylated DNA in a background of unmethylated DNA (see Table S5). For all primer combinations, with exception of ACTB, no fragments were amplified with unmethylated, background DNA.

### Statistical analysis

Explorative data analyses were performed for all three samplings. The proportion of methylation-positive test results was calculated according to the histologically confirmed cervical disease status. Additionally, in the cross-sectional study on outpatients attending our colposcopy clinic sensitivity and specificity were estimated along with exact 95% confidence intervals (CI) assuming a binomial distribution. The test was scored positive if at least two of five markers were methylated. The estimates were given for CIN2+ and CIN3+ cut-off points with and without cancer cases, respectively. Proportions were statistically compared by the Fisher exact test, the level of significance was set to 0.05. To address the effect of spectrum bias, test performance for detection of CIN2+ and CIN3+ was further projected to the target population of a HPV screening study which we had performed in Eastern Thuringia some years ago [20]. Now, sensitivity, specificity and predictive values were calculated by weighting the methylation rates from sample 3 according to the distribution of the disease status observed in that target population.

## Supporting Information

Figure S1 **Average Ct values for all methylation positive cases (sampling 3) according to histological classification and age.**
(DOCX)

Figure S2 **Representative melting curves for each marker.**
(DOCX)

Table S1 **Candidate CpG islands analysed with qMSP.**
(DOCX)

Table S2 **HPV genotyping of CIN3 and cancer cases of sampling 1.**
(DOCX)

Table S3 **Excel file comprising methylation scores of all markers for sampling 3.**
(XLSX)

Table S4 **HPV genotyping of all CIN and cancer cases of sampling 3.**
(DOCX)

Table S5 **Sensitivity and Specificity of qMSP demonstrated in a serial dilution of in-vitro methylated, bisulfite-treated DNA in a background of bisulfite-treated DNA from a cervical scrape of a healthy woman.**
(DOC)
