# Supplementary material for: A Promising DNA Methylation Signature for the Triage of High-Risk Human Papillomavirus DNA-Positive Women
Source: PLoS One. 2014 Mar 19;9(3):e91905. doi: 10.1371/journal.pone.0091905 (PMC3960142; doi:10.1371/journal.pone.0091905)
Supplement: Figure S1 — Average Ct values for all methylation positive cases (sampling 3) according to histological classification and age. (DOCX) [file pone.0091905.s001.docx]

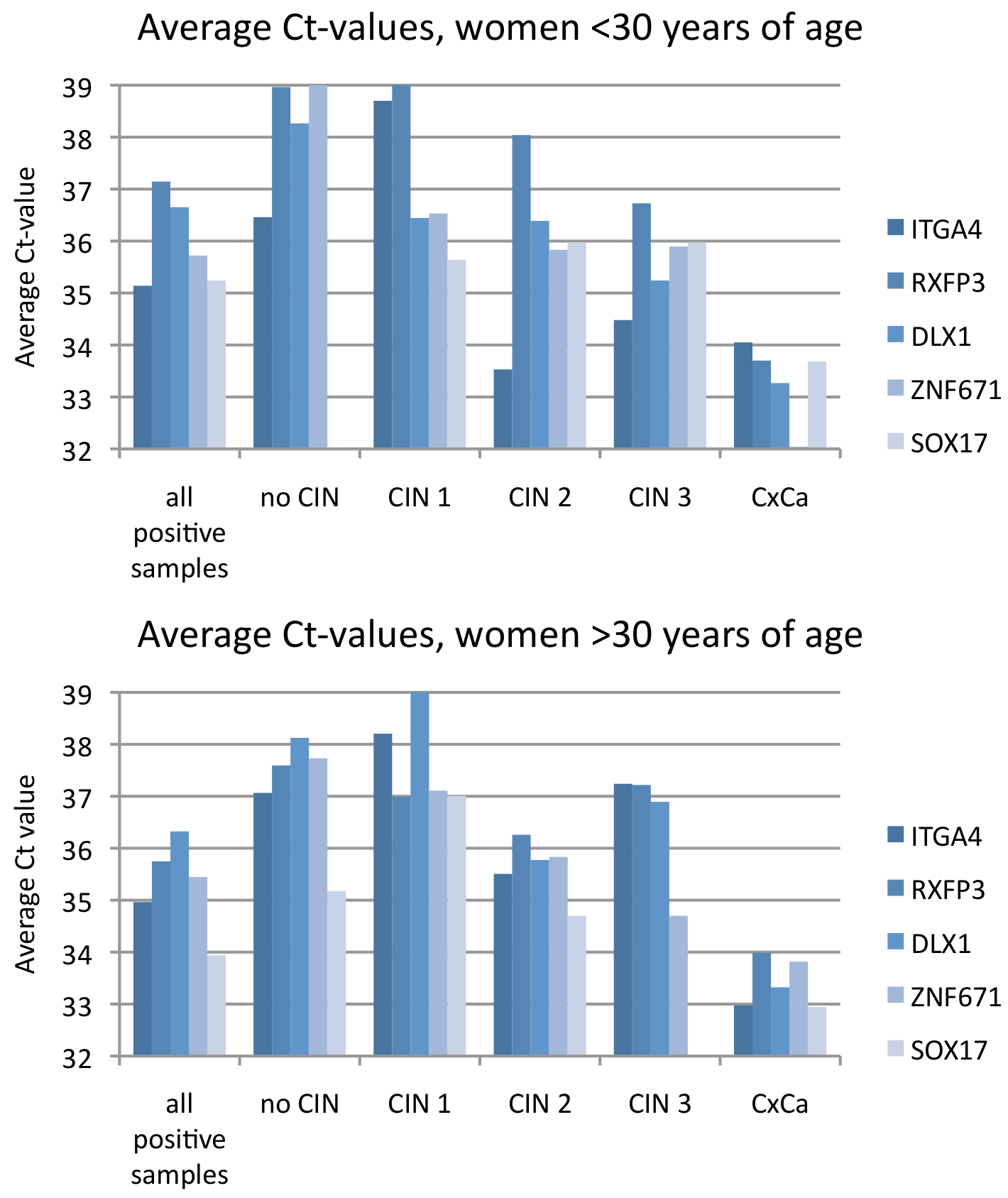


Supplementary Figure S1. Average Ct-values obtained for all MSP reactions scored methylation-positive in sampling 3.
