# Supplementary material for: A Promising DNA Methylation Signature for the Triage of High-Risk Human Papillomavirus DNA-Positive Women
Source: PLoS One. 2014 Mar 19;9(3):e91905. doi: 10.1371/journal.pone.0091905 (PMC3960142; doi:10.1371/journal.pone.0091905)
Supplement: Figure S2 — Representative melting curves for each marker. (DOCX) [file pone.0091905.s002.docx]

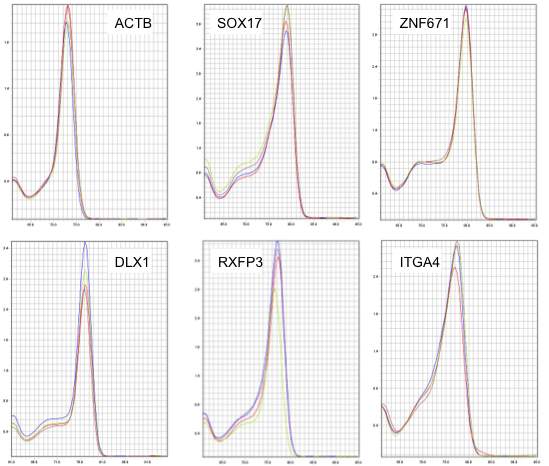


Supplementary Figure S2: Four melting curves each are shown for all five markers and the positive control ACTB. The melting curves are representative for positive MSP of cervical carcinoma (blue), CIN3 (red), CIN2 (violet), CIN1 (green). On the x-axis, the temperature in °C is shown, on the y-axis, the derivative reporter (normalized to ROX reference dye) is shown. The melting points of the PCR products are: ACTB:73.1°C, SOX17: 79.0°C, ZNF671: 79.8°C, DLX1: 77.1°C, RXFP3: 77.0°C, ITGA4: 77.1°C.
