# Supplementary material for: A Promising DNA Methylation Signature for the Triage of High-Risk Human Papillomavirus DNA-Positive Women
Source: PLoS One. 2014 Mar 19;9(3):e91905. doi: 10.1371/journal.pone.0091905 (PMC3960142; doi:10.1371/journal.pone.0091905)
Supplement: Table S1 — Candidate CpG islands analysed with qMSP. (DOCX) [file pone.0091905.s003.docx]

Supplementary Table S1: Candidate CpG islands analysed with qMSP

| AGTR1 | FBXO18 | LHX9 | PAK3 | RYR2 |
| --- | --- | --- | --- | --- |
| ASTN1 | FGF12 | LMX1A | PAX1 | SFRP2 |
| BAI2 | FGF17 | LRRTM1 | PAX5 | SHOX |
| BCAN | FLRT2 | MAB21L1 | PAX9 | SLITRK1 |
| CACNA1E | FOXD2 | MAP9 | PCDH10 | SOX17 |
| CACNG7 | FOXG1 | MEF2C | PCDH17 | SSTR1 |
| CCDC111 | GDNF | MLLT1 | PCDHAC1 | TCHH |
| CFHR4 | GHSR | MPO | PDE4DIP | TMC2 |
| CNTN5 | GRIN2D | MYBPC1 | PDGFD | TRAM1L1 |
| CNTNAP2 | HOXD10 | MYF6 | PEX5L | TRDMT1 |
| CYP4V2 | IGSF22 | NEFM | PFKFB31 | TRH |
| DBC1 | IL15RA | NELL1 | PFKFB32 | TRPA1 |
| DGKI | INSRR | NID2 | PGLYRP1 | TUBB4Q |
| DLX1 | IRF1 | NLGN1 | PRKCQ | UBNX |
| DMRTA2 | IRX1 | NPHS1 | PROX1 | WDR37 |
| EDIL3 | ITGA4 | NPY | PTGER2 | WDR52 |
| EDN3 | ITGB7 | NXPH1 | RAB6C | WNT4 |
| EML1 | KCNA1 | OGDHL | REC8 | ZIM2 |
| EVX2 | KCNN2 | OSBPL2 | RIMS1 | ZNF215 |
| FAM78B | KRT72 | OXCT2 | RXFP3 | ZNF671 |

Of 100 CpG regions identified by microarray analysis 24 CpG regions highlighted in colour showed promise when comparing pooled DNAs of scrapes from HPV-positive women without cervical disease and scrapes from women with cervical carcinoma by qMSP. These regions were further analysed using single cervical scrapes. The regions highlighted in yellow could discriminate best. The regions highlighted in blue are potential candidates for further validation.
