# Supplementary material for: A Promising DNA Methylation Signature for the Triage of High-Risk Human Papillomavirus DNA-Positive Women
Source: PLoS One. 2014 Mar 19;9(3):e91905. doi: 10.1371/journal.pone.0091905 (PMC3960142; doi:10.1371/journal.pone.0091905)
Supplement: Table S2 — HPV genotyping of CIN3 and cancer cases of sampling 1. (DOCX) [file pone.0091905.s004.docx]

| HPV-type | | | | | | | | | |
| --- | --- | --- | --- | --- | --- | --- | --- | --- | --- |
|  | 16 | 18 | 31 | 33 | 45 | 52 | 58 | other HR-type | number of cases |
| CxCa |  |  |  |  |  |  |  |  | Σ 54 |
| Methylation positive | 49 | 5 | - | - | - | - | - | - | 54 |
| Methylation negative | - | - | - | - | - | - | - | - | 0 |
| CIN3 |  |  |  |  |  |  |  |  | Σ 43 |
| Methylation positive | 18 | 1 | 3 | 2 | - | - | - | 2 | 24 |
| Methylation negative | 11 | - | 3 | - | - | - | - | 6 | 19 |

Supplementary Table S2: HPV genotyping of CIN3 and cancer cases of sampling 1

Double infections count for each HPV type, so number of biopsies may be lower than total number of HPV types. To be scored as „methylation positive“ at least 2 of 5 markers were required to be methylated.
