# Supplementary material for: A Promising DNA Methylation Signature for the Triage of High-Risk Human Papillomavirus DNA-Positive Women
Source: PLoS One. 2014 Mar 19;9(3):e91905. doi: 10.1371/journal.pone.0091905 (PMC3960142; doi:10.1371/journal.pone.0091905)
Supplement: Table S4 — HPV genotyping of all CIN and cancer cases of sampling 3. (DOCX) [file pone.0091905.s006.docx]

| HPV-type | | | | | | | | | |
| --- | --- | --- | --- | --- | --- | --- | --- | --- | --- |
|  | 16 | 18 | 31 | 33 | 45 | 52 | 58 | other HR-type | number of cases |
| CxCa |  |  |  |  |  |  |  |  | Σ 19 |
| ≥ 30 years |  |  |  |  |  |  |  |  | 17 |
| Methylation positive | 11 | 1 | 1 | 1 | - | - | - | 4 | 17 |
| Methylation negative | - | - | - | - | - | - | - |  | 0 |
| < 30 years |  |  |  |  |  |  |  |  | 2 |
| Methylation positive | 2 | - | - | - | - | - | - | - | 2 |
| Methylation negative | - | - | - | - | - | - | - | - | 0 |
| CIN3 |  |  |  |  |  |  |  |  | Σ 23 |
| ≥ 30 years |  |  |  |  |  |  |  |  | 9 |
| Methylation positive | 5 | - | - | 1 | 1 | 1 | - | - | 8 |
| Methylation negative | - | 1 | - | - | 1 | - | - | - | 1 |
| < 30 years |  |  |  |  |  |  |  |  | 14 |
| Methylation positive | 4 | - | 1 | - | - | - | - | - | 5 |
| Methylation negative | 4 | - | 2 | 1 | - | - | - | 2 | 9 |
| CIN2 |  |  |  |  |  |  |  |  | Σ 42 |
| ≥ 30 years |  |  |  |  |  |  |  |  | 13 |
| Methylation positive | 5 | 1 | - | 1 | - | - | - | 3 | 9 |
| Methylation negative | 3 | - | - | - | - | - | - | 1 | 4 |
| < 30 years |  |  |  |  |  |  |  |  | 29 |
| Methylation positive | 5 | - | 1 | - | - | - | - | - | 6 |
| Methylation negative | 13 | 4 | 3 | 2 | 2 | 2 | 2 | 1 | 23 |
| CIN1 |  |  |  |  |  |  |  |  | Σ 28 |
| ≥ 30 years |  |  |  |  |  |  |  |  | 10 |
| Methylation positive | 3 | - | - | - | - | - | - | - | 3 |
| Methylation negative | 2 | 1 | 3 | - | - | - | - | 1 | 4 |
| < 30 years |  |  |  |  |  |  |  |  | 18 |
| Methylation positive | 1 | - | - | - | - | - | - | - | 1 |
| Methylation negative | 10 | - | 3 | - | - | - | 1 | 3 | 17 |

Supplementary Table S4: HPV genotyping of all CIN and cancer cases of sampling 3

Double infections count for each HPV type, so number of biopsies may be lower than total number of HPV types. To be scored as „methylation positive“ at least 2 of 5 markers were required to be methylated.
