# Supplementary material for: A Promising DNA Methylation Signature for the Triage of High-Risk Human Papillomavirus DNA-Positive Women
Source: PLoS One. 2014 Mar 19;9(3):e91905. doi: 10.1371/journal.pone.0091905 (PMC3960142; doi:10.1371/journal.pone.0091905)
Supplement: Table S5 — Sensitivity and Specificity of qMSP demonstrated in a serial dilution of in-vitro methylated, bisulfite-treated DNA in a background of bisulfite-treated DNA from a cervical scrape of a healthy woman. (DOC) [file pone.0091905.s007.doc]

Supplementary Table S5: Sensitivity and Specificity of qMSP

|  | 100% | | 25% | | 10% | | 5% | | 2% | | 1% | | 0% | |
| --- | --- | --- | --- | --- | --- | --- | --- | --- | --- | --- | --- | --- | --- | --- |
| marker | Mean | +/- | Mean | +/- | Mean | +/- | Mean | +/- | Mean | +/- | Mean | +/- | Mean | +/- |
| ACTB | 29,31 | 0,13 | 31,4 | 0,22 | 32,24 | 0,02 | 32,21 | 0,64 | 31,82 | 0,12 | 32,49 | 0,28 | 32,9 | 0,32 |
| DLX1 | 32,47 | 1,05 | 33,18 | 0,26 | 36,04 | 1,54 | 36,98 | 0,34 | 38,36 | 0,55 | – | – | – | – |
| ITGA4 | 26,68 | 0,09 | 28,67 | 0,29 | 30,42 | 0,37 | 31,65 | 0,64 | 32,97 | 0,18 | 33,89 | 0,12 | – | – |
| RXFP3 | 27,07 | 0,18 | 29,41 | 0,03 | 31,35 | 0,37 | 32,4 | 0,74 | 33,45 | 0,35 | 34,92 | 0,07 | – | – |
| SOX17 | 28,17 | 0,37 | 29,37 | 0,11 | 31,05 | 0,46 | 32,16 | 0,44 | 33,36 | 0,49 | 35,06 | 0,48 | – | – |
| ZNF671 | 29,45 | 0,72 | 31,26 | 0,34 | 32,42 | 0,17 | 34,08 | 0,6 | 34,73 | 0,29 | 36,71 | 0,43 | – | – |

Mean Ct values obtained for all markers for a serial dilution of in-vitro methylated, bisulfite-treated DNA in a background of bisulfite-treated DNA from a cervical scrape of a healthy woman (100% to 0%). qMSP was done in triplicate. Standard deviations are given as +/-. Only Ct values with correct melting curves are shown.” – “ refers to no amplification. No PCR-products were obtained with bisulfite-treated DNA from a cervical scrape of a healthy woman for any of the markers with exception of ACTB.
